# Increasing the performance of pooled CRISPR–Cas9 drop-out screening

**DOI:** 10.1038/srep31782

**Published:** 2016-08-22

**Authors:** Benedict C. S. Cross, Steffen Lawo, Caroline R. Archer, Jessica R. Hunt, Joanne L. Yarker, Alessandro Riccombeni, Annette S. Little, Nicola J. McCarthy, Jonathan D. Moore

**Affiliations:** 1 Horizon Discovery, 8100 Cambridge Research Park, Waterbeach, Cambridge, CB25 9TL, United Kingdom

## Abstract

Components of the type II CRISPR–Cas complex in bacteria have been used successfully in eukaryotic cells to facilitate rapid and accurate cell line engineering, animal model generation and functional genomic screens. Such developments are providing new opportunities for drug target identification and validation, particularly with the application of pooled genetic screening. As CRISPR–Cas is a relatively new genetic screening tool, it is important to assess its functionality in a number of different cell lines and to analyse potential improvements that might increase the sensitivity of a given screen. To examine critical aspects of screening quality, we constructed ultra-complex libraries containing sgRNA sequences targeting a collection of essential genes. We examined the performance of screening in both haploid and hypotriploid cell lines, using two alternative guide design algorithms and two tracrRNA variants in a time-resolved analysis. Our data indicate that a simple adaptation of the tracrRNA substantially improves the robustness of guide loss during a screen. This modification minimises the requirement for high numbers of sgRNAs targeting each gene, increasing hit scoring and creating a powerful new platform for successful screening.

Clustered regularly interspaced short palindromic repeats (CRISPRs) form part of an adaptive immune response in bacteria that is activated by the presence of foreign DNA elements from virus or plasmids^1^,^2^. Recognition of these genetic elements triggers the assembly of a protein–RNA complex, similar to RNA interference complexes in eukaryotes, which cleaves the invading DNA. Components of this complex — CRISPR associated (Cas) nuclease, CRISPR RNA (crRNA) and a trans-activating crRNA (tracrRNA) — have been modified and used in eukaryotic cells to facilitate rapid and accurate cell line engineering and animal model generation, as well as functional genomic screens^3^. Such developments provide new opportunities for drug target identification and validation, particularly in the context of a pooled genetic screening format.

In eukaryotic cells, expression of Cas9 concomitant with an sgRNA, which comprises a crRNA and a tracrRNA, leads to the generation of a protein–RNA complex that can bind to DNA at a locus defined by the sgRNA. Cas9 induces DNA double strand breaks three base pairs up-stream of a protospacer adjacent motif (PAM) site, which is present in the target DNA sequence. The DNA double strand break is primarily repaired by non-homologous end joining (NHEJ) repair mechanisms, resulting in the introduction of insertions or deletions (InDels) and potential disruption of the locus of interest^4^. When the sgRNA targets early coding exons, this can lead to disruption of the target open reading frame (ORF) and a lack of stable or complete mRNA, resulting in a gene knockout. CRISPR-based screening has shown early promise and appears to have some advantages over existing screening technologies, such as shRNA and siRNA^5^,^6^,^7^.

Pooled CRISPR screens using lentiviral transduction to deliver both the Cas9 nuclease and sgRNAs into cells enable the effect of knocking out thousands of individual genes to be assessed in a phenotypic readout, such as proliferation or cell death. The screen analysis is based on data from cells collected at the start of the screen (3–5 days after transduction) compared with cells collected at the end of a screen that have been exposed to a drug of interest, for example. The next generation sequencing (NGS) data from both the initial time point and the initial library acts as a comparator for the NGS data collected at the end of the screen, such that sgRNA loss and gain over the time of the screen can be established. These data can also be assessed using algorithms such as MAGeCK^8^ and BAGEL^9^, which enables individual sgRNAs and gene hits to be ranked. Most published screens have looked at resistance to drug treatment, where the loss of function at a resistance-inducing locus will result in the accumulation of that population of mutagenised cells. By contrast, sensitivity screens rely on loss of specific guide RNAs from the initial starting population as a means to identify genes, the loss of which increases sensitivity of the cells to a drug (or genotype) of interest. These screens in particular provide innate challenges due to the limit of sensitivity of both the mutagenesis and detection technology, and these challenges are amplified for whole-genome screens where loss of individual guides or genes can be difficult to discern above the noise of the experiment. In addition, target identification screens in cancer cell lines also have to contend with poly-ploidy and aneuploidy, both of which impact screening optimisation and statistical power.

As the CRISPR–Cas technology is a relatively new genetic screening tool, it is important to assess its functionality in a number of different cell lines and to analyse potential improvements that might increase the sensitivity of a given screen. To examine the effects on drop-out screens of guide number and tracrRNA modifications, we constructed a high complexity library containing sgRNA sequences targeting a collection of known essential genes. We included guides from a second generation (2G) guide design system (GeCKOv2 collection^10^) and the machine-learned set from the Lander and Sabatini laboratories (third generation (3G) guide design^11^). We also included a large collection of inactive guides identified in a whole-genome screen in HL-60 cells^11^ and dummy guides^10^ to provide neutral controls. Our results indicate that the number of guides per target gene can impact the sensitivity of drop-out screens in both haploid and triploid cell lines. However, alterations to the tracrRNA that increase the residency time of Cas9 and remove a potential pol III stop site^12^, further improve the sensitivity of drop-out screens and essentially negate the effect of numbers of sgRNAs targeting each target gene.

## Results

**Figure 1:**
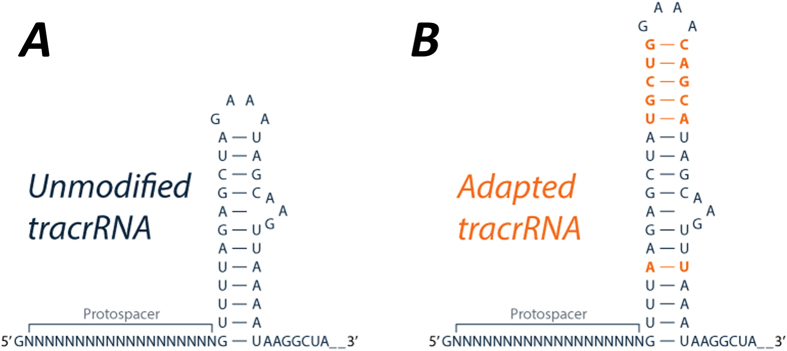
Adaptation of tracrRNA sequence to improve CRISPR–Cas9 knock-out screening. (**A**) The sequence of the original tracrRNA from the fusion molecule^18^ and found in pLentiCRISPRv2^13^. (**B**) The adapted tracrRNA providing apparent increased residency time of the Cas9 and target DNA^12^.

We generated two pooled guide RNA libraries against the same 100 essential genes. This comprised 70 essential ribosomal genes, 10 genes found to be essential for viability in HL60 cells (Group A^11^), and 20 essential genes that overlap between KBM7 and HL60 cells (Group B^11^). In total, 16 sgRNAs against each essential gene were captured in the library, including 10 guides per gene from a third generation guide design (machine learned guides from Wang *et al*.^11^) and 6 guides per gene from the algorithm used by Feng Zhang and colleagues^10^. Ten guide RNAs targeting 750 putatively neutral genes^11^ and 1000 non-targeting guides^10^ were also included. One version of the sgRNA library was produced using the chimeric tracrRNA sequence as used by Shalem *et al*.^13^. The second version of this library was produced using an adapted chimeric tracrRNA sequence in which a potential pol III termination site was knocked out and the Cas9 binding hairpin structure was extended, as described in Chen *et al*., 2013 (Fig. 1)^14^. In either case, the a single vector system that expresses both Cas9 and an sgRNA^13^ was used and lentiviral particles generated using HEK293T cells.

Two cell lines were then transduced with the libraries: the fully haploid eHAP line (engineered from HAP1 cells lines, which are derived from the near haploid leukaemia line KBM7. See Supplementary Figure S1)^15^ and the hypotriploid A375 melanoma cell line. As the library targets essential genes, loss of expression of these genes will be expected to result in the death and loss of these cells and their resident guides from the population during the screen. The temporal properties of guide depletion were assessed at each of four time points (T_1_ − T_4_) collected throughout the screen.

**Figure 2:**
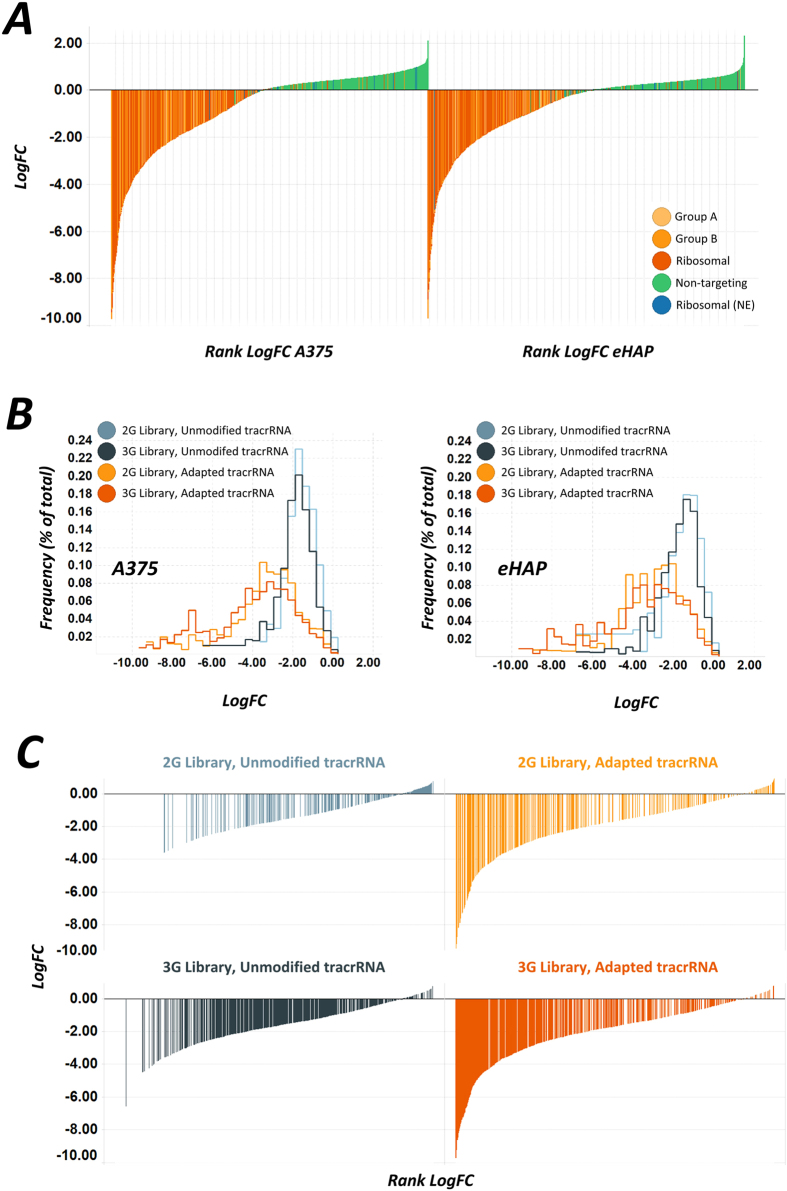
Evaluation of CRISPR–Cas9 Screening in haploid and hypotriplod cell lines reveals distinct responses in screen performance using an adapted tracrRNA sequence. (**A**) Log_2_ fold change (LogFC) of control guides in the screening for each cell line is shown determined between T1 and T3 samples (see methods). Guides targeting essential genes are shown in orange, and negative control guides are shown in blue and green. Genes within each group are shown in Table S1 (Supplementary Information) (**B**). Distribution plots of essential guides in each library (2G and 3G) and each tracrRNA variant (unmodified and adapted), showing the LogFC as a percentage of the guides in each group. Inactive guides score close to zero, and are found with lower frequency in the adapted tracrRNA screen and in the 3G library. (**C**) Waterfall plots for individual guide LogFC scores in A375 cells plotted for each library and each tracrRNA variant showing the rank of each guide within each set for side-by-side comparison of the distributions within each dataset.

Both cell lines showed a clear partition of positive and negative control guides, with a similar distribution where essential guide depletion was measured up to 1000-fold over 12 population doublings (Fig. 2A). A direct comparison of guide performance in both A375 and eHAP cells using the unmodified tracrRNA library indicated that the third generation guides had the greatest efficacy, as a proportionally greater number of guides showed high levels of drop-out (Fig. 2B,C). Additionally, the third generation guide collection contained fewer inactive guides in the essential gene groups, supporting the value of the machine learned guide selection process and the increased number of guides per gene in this library (Fig. 2B,C). Strikingly, both guide sets were found to perform approximately equally and substantially better when using the adapted tracrRNA for gene knock-out screening (Fig. 2B,C). This modification resulted in a substantially greater number of guides dropping out to a very high magnitude during the screen and fewer inactive guides than the unmodified counterpart screen.

**Figure 3:**
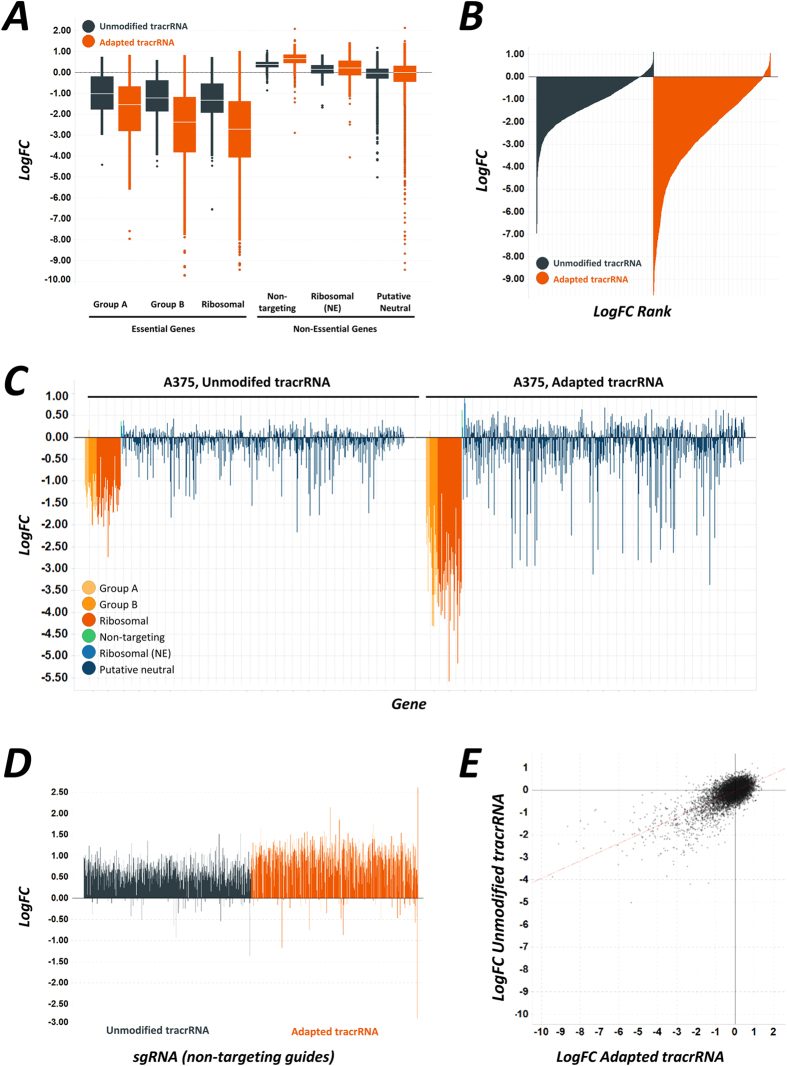
Screening using adapted tracrRNA sequence provides a substantial increase in the sensitivity of CRISPR–Cas9 screening. (**A**) Box plots showing median guide LogFC for each group of genes and in each tracrRNA variant. In the case of essential guide groups, the adapted tracrRNA results in substantially improved drop-out rates. (**B**) Waterfall plots for the essential guides shown for each tracrRNA variant in A375 cells. (**C**) Mean LogFC drop-out rates for each gene in the A375 screen for each tracrRNA variant, separated into groups. (**D**) LogFC values for the non-targeting guides in each screen in A375 cells. (**E**) Comparison of the LogFC between the two tracrRNA variants for the putative neutral guides.

**Figure 4:**
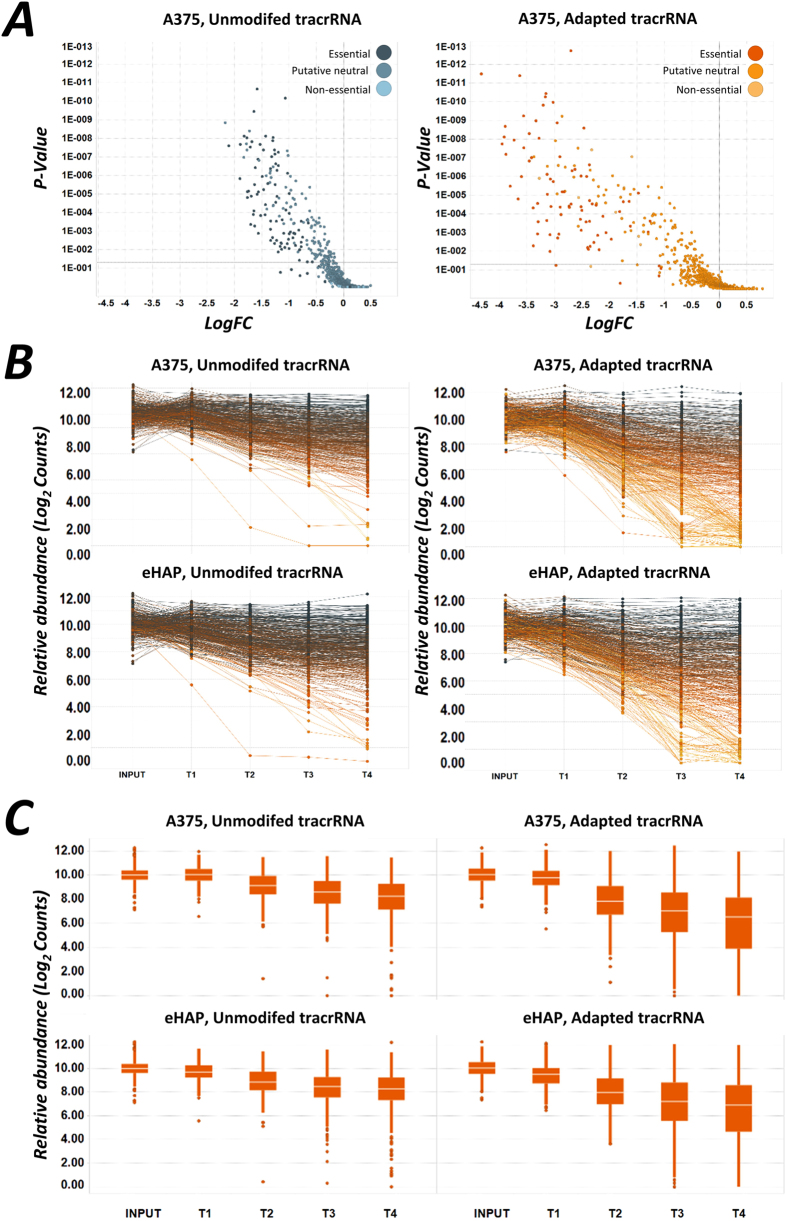
Kinetic analysis of CRISPR–Cas9 screening indicates that gene essentially can be manifested at an early time-point following infection with Cas9. (**A**) Volcano plots from one-tailed analysis using the RRA analysis (p-value) algorithm for each of the genes in the two tracrRNA datasets^8^. (**B**) Comparison of the relative abundance in Log2 sequence counts at each time point measured for essential gene guides in each group. Colours indicate the LogFC for each guide from, where yellow is the maximum and dark blue in the minimum. T1 samples were taken after 72 hours after transduction, T2 after seven days, T3 was taken 12 doublings after T2 and T4 was taken 24 doublings after T2. (**C**) Box plots for the time-resolved data shown in A, indicating that no major overall difference in screen characteristics was observed between eHAP and A375 cells.

Further analyses of these data from A375 cells indicates that the modified tracrRNA leads to a substantially increased drop out of guides targeting essential genes at the functional group level (Fig. 3A), guide level (Fig. 3B) and at the gene level (Fig. 3C). Many of the putatively neutral genes identified in HL60 cells were found to impact the survival of A375 cells, as shown by the substantial drop-out of some of these guides during the screen (Figs 3A and 2C). This reflects that for some genes, their essentiality is potentially cell line or tissue type dependent^16^. Drop-out rates for these putative genotype-dependent lethal genes were found to be greater when using the adapted tracrRNA (Fig. 3C), reflecting the increase in efficacy resulting from this modification. Importantly, gene essentiality, when it occurred, was observed in both screening systems, albeit without a strong guide-by-guide correlation (Fig. 3C,E). Very similar trends were observed when the same analysis was conducted in haploid cells (Supplementary Figure S2). The non-targeting guides were found to behave similarly in each screening system (Fig. 3D). These guides show modest enrichment over the duration of the screen, presumably because no DNA damage is enacted by the non-targeting guide–Cas9 complex. Any increase in cytotoxic effects might be indicative of an increase in off-target consequences owing to the adapted tracrRNA. By contrast, these guides were found to enrich to a greater extent when the adapted tracrRNA was used (cf. Fig. 3A,D). This is most likely a consequence of the commensurate decrease in complexity in the cellular population due to the increased drop-out of essential guide-bearing clones. Thus, the increased loss of essential guides from the adapted tracrRNA population allows for a greater accumulation of the competitively-advantaged non-targeting guides. Analysis of the overall gene-level hit scoring for essential gene indicated that RRA scores (p-values) from the adapted tracrRNA were improved compared with the unmodified tracrRNA, as expected (Fig. 4A).

To assess drop-out kinetics and reproducibility, we compared the performance of the guides targeting essential genes over multiple time points in eHAP and A375 cells. These data indicate that loss-of-viability owing to editing by CRISPR–Cas9 can occur rapidly, and that at an early time point in the screen (T2, seven days after selection initiation), a substantial number of guides had already been depleted from the population (Fig. 4B). Guide depletion was more pronounced and more rapid when using the adapted tracrRNA, and the maximum magnitude of drop-out was greater in either cell line when using this system (Fig. 4B). These data are well associated across both libraries between each cell line indicating excellent reproducibility. Surprisingly, drop-out rates and kinetics were found to be relatively similar in either the haploid or hypotriploid cell line (Fig. 4B,C). This indicates that CRISPR–Cas9 screening is able to enact lethal levels of gene depletion even in cell lines with multiple alleles and might therefore suggest a high incidence of homozygous disruption in these screens.

## Discussion

We have used a high complexity sgRNA screen to evaluate guide design algorithms and the impact of tracrRNA modification in haploid and triploid cell lines. These analyses indicate that guide RNA algorithms can impact the robustness of the data generated in drop-out sensitivity screens. However, as the third generation guide set contains up to ten guides per gene compared with the six guides per gene in the second generation GeCKOv2 library, this might also represent a sampling bias in favour of the third generation guide set. Nevertheless, this apparent impact is largely negated when an adapted tracrRNA is used which provides a substantial increase in performance and approximately equilibrates the performance of the two guide sets. Use of the adapted tracrRNA substantially increases the number of guides lost during the duration of the screen, the speed of guide loss over time and the percentage of guides being lost by the end of the screen. This impact was evident in both haploid and hypotriploid cell lines.

The tracrRNA modifications used in our screens were initially described by Chen and colleagues^14^ to improve the use of CRISPR–Cas9 for imaging purposes. Using a nuclease-deficient form of Cas9 (known as dCas9), Chen and colleagues showed that modifying the tracrRNA such that it was closer to the form present in bacteria, substantially improved the binding of enhanced green fluorescent protein (EGFP)–dCas9 fusion protein to telomeres. Our data indicate for the first time that the same modification of the tracrRNA also improves the function of nuclease proficient Cas9 in a pooled screening format. Adaptation of the tracrRNA sequence appears to increase the residency time for the holoenzyme complex^14^, presumably providing a corresponding increase in the frequency of catalysis and double strand breakage by Cas9. This discovery should therefore be broadly applicable to any activity in which Cas9 editing is required.

The increased binding of Cas9 to the target site of interest might have potentially impacted the off-target effects of Cas9, manifested through an increase in binding to mis-matched DNA sequences. However, in a whole genome or complex library screening format, the impact of these off-target effects are unlikely to substantially impact the screen readout on a gene by gene basis. Moreover, although we have not directly monitored off-target activity here, we did not find any evidence for an increase in off-target effects, since genes showed similar behaviour in either screening system and no increase in potential off-target effects was detected for non-targeted guides expressed with either tracrRNA variant. Further detailed analysis of the mechanism for this improved tracrRNA sequence would also help to explore any off-target consequences arising from the modification.

Our data indicate that a simple adaptation of the tracrRNA substantially improves the robustness of guide loss during a screen. Application of this discovery will commensurately increase the chances of identifying new targets that have eluded existing functional genomic screening platforms. Although we have used sensitivity as a vehicle to evaluate these aspects of screening, these observations should be equally applicable to multiple formats for screening with CRISPR–Cas9, including resistance screening, reporter screening and synthetic lethal target discovery. Thus, when deployed astutely with diligent validation of potential targets, CRISPR–Cas9 screens offer researchers an improved and rapid insight into complex biological problems.

## Methods

### Cell lines

eHAP cells (Horizon Discovery Ltd, UK) were grown in IMDM supplemented with 10% FBS and 2 mM L-glutamine (all supplied by Gibco, UK). A375 melanoma cells (ATCC, USA) were thawed and cultured in DMEM supplemented with 10% FBS and 2 mM L-glutamine (all supplied by Gibco, UK). Cells were routinely checked for mycoplasma and identity verified by STR analysis.

### Library generation

We generated two pooled guide RNA libraries against 100 essential genes using three different guide RNA design algorithms: the machine learning based guides generated by Lander and Sabatini’s group^11^ (10 sgRNAs per target gene); and the second generation guides used by the Zhang laboratory^10^ (6 sgRNAs per target gene). Included in the pooled library, but not analysed or discussed here, were guides designed by a Desktop Genetics–Horizon Discovery algorithm (variable numbers of sgRNAs per target). In addition, 10 guide RNAs targeting 750 neutral genes using the 3G algorithm^11^ were included. Each sgRNA was synthesised twice (Custom Array Inc); once to allow compatibility with the tracrRNA sequence used in the original GeCKOv2 library (5′-GTTTTAGAGCTAGAAATAGCAAGTTAAAATAAGGC-3′)^10^ and once to allow compatibility with the modified tracrRNA sequence as described in Chen *et al*., 2013 (5′-GTTTAAGAGCTA*TGCTG*GAAA*CAGCA*TAGCAAGTT-3′)^14^. An all-in-one lentivirus plasmid vector was built comprising a selection marker (puromycin resistance), the expression cassette for SpCas9 expression and the sgRNA sequence (based on pLentiCRISPRv2^10^). This backbone was then mutagenised to adapt the tracrRNA sequence in the plasmid to match that used for GFP labelling with dCas9 fusions^14^, yielding pLentiCRISPR_HZD01.

Each pooled sgRNA library was cloned into either vector backbone, using a Gibson Assembly Master Mix kit (New England BioLabs, NEB #E2611S/L) in accordance with the manufacturer’s instructions. Library plasmids were purified using a Qiagen Plasmid Plus purification system in accordance with the manufacturer’s instructions.

### Lentivirus production

HEK293T cells (ATCC, USA) were grown in DMEM and 10% FBS (Gibco, UK). 24 hours ahead of transfection with the library vectors, HEK293T cells were seeded into T225 flasks (Corning) at 40% confluency. The following day, the cells were transfected with the library plasmids using Lipofectamine 3000 (Invitrogen, USA) and Virapower packaging virus (LifeTechnologies, UK) in accordance with the manufacturer’s instructions. Briefly, the medium was removed from the HEK293T cells and the DNA–lipid mix was added to the cells in Optimem medium (Gibco, UK) and left for 6 hours, after which the transfection mix was removed and replaced with DMEM containing 10% FBS and 1% BSA (Sigma-Aldrich). The medium was harvested 48 hours later and centrifuged at 500 × g for 10 min at 4 °C and then filtered using a 0.45 μm low protein-binding filter (Millex Durapore PVDF HF). The virus was further concentrated using Lenti-X concentrator (Clontech #631232) in accordance with the manufacturer’s instructions. The viral supernatant was aliquoted and stored at −80 °C in DMEM with 10% FBS and 1% BSA.

### Cell transduction and screening protocol

Functional titration was used to identify the transduction conditions that allow use of a low MOI (~0.3) at which the majority of cells are infected with a single viral particle. This is particularly important for screening where the generation of single knockouts is the main goal. Once each lentiviral library had been functionally titrated in both eHAPs and A375 cells, the cells were trypsinized, seeded in complete medium supplemented with 0.8 ug/ml polybrene (Sigma-Aldrich) and transduced with each library according to standard lentiviral transduction protocols. Briefly, cells were seeded into 12 well dishes at 2 × 10^6^ cells per well and spinfected for 2 hours at 2000 rpm at 37 °C using virus diluted to achieve an MOI of 0.3. A T175 cm^2^ flask of untransfected cells was also set up for each cell line. The cells from all 24 spinfected wells were resuspended, transferred to a 50 ml falcon and centrifuged at 1000 rpm for 5 minutes. The supernatant was removed and cells were resuspended in 50 ml fresh media (without polybrene) and transferred to four T175 cm^2^ flasks at 12 × 10^6^ cells per flask per library per line. 24 hours later the transduced and non-transduced cells were treated with puromycin at a final concentration of 0.4 μg/ml. 72 hours after addition of puromycin, 3 technical replicate pellets of 30 × 10^6^ cells were harvested and flash frozen from each cell line transduced with each library (T_1_). The remaining cells were maintained in puromycin in 5 layer flasks (Falcon) at 12 × 10^6^ cells per flask. The cells were counted and reseeded at 12 × 10^6^ per 5 layer flask every 2–3 days and puromycin selection was stopped 8 days after spinfection when all non-transduced cells were dead. The cells were passaged for a total of 4 weeks and additional pellets were harvested 7 days post puromycin addition (T_2_), 12 doublings after the T_2_ pellet (T_3_) and 24 doublings after the T_2_ pellet (T_4_). All frozen pellets were thawed and gDNA extracted using Promega Maxwell. DNA concentration was determined using a Nanodrop spectrophotometer and samples were then subjected to PCR to generate amplicons of the sgRNA cassette^13^ using a forward primer: TCGTCGGCAGCGTCAGATGTGTATAAGAGACAGU–[Variable]–TGTGGAAAGGACGAAACACC; and a reverse primer: GTCTCGTGGGCTCGGAGATGTGTATAAGAGACAGGATCAATTGCCGACCCCTCC. These amplicon samples were purified using Agencourt beads (Beckman) and deep sequenced on an Illumina NextSeq (Microsynth AG, Switzerland).

### Data analysis

Raw NGS libraries were evaluated for quality using FASTQC version 0.11.5. (Babraham Institute, Cambridge UK)^17^. All libraries passed QC. Guide counts were obtained using an in-house customized version of the MAGeCK workflow version 0.5.0^8^, which took into account guide staggering from the experimental protocol. Briefly, guides were trimmed and mapped with exact string counts from each file to provide raw counts for each guide found in the library. Guide counts were normalised within each group (median-based) and Log_2_ fold change (LogFC) was calculated to determine the change in abundance of each guide in each sample. RRA values (p-values) were determined using the MAGeCK algorithm (version 0.5.0), as described in Li *et al*.^8^. Unless otherwise indicated, LogFC is determined between the early timepoint (T1) and 12 population doublings (T3).

## Additional Information

**How to cite this article**: Cross, B. C. S. *et al*. Increasing the performance of pooled CRISPR–Cas9 drop-out screening. *Sci. Rep.* **6**, 31782; doi: 10.1038/srep31782 (2016).

## Supplementary Material

Supplementary Information
