# Supplementary material for: Increasing the performance of pooled CRISPR–Cas9 drop-out screening
Source: Sci Rep. 2016 Aug 22;6:31782. doi: 10.1038/srep31782 (PMC4992892; doi:10.1038/srep31782)
Supplement: Supplementary Information [file srep31782-s1.pdf]

SUPPLEMENTAL INFORMATION FOR:

Increasing the performance of pooled CRISPR–Cas9 drop-out screening

Benedict C.S. Cross*[*], Steffen Lawo, Caroline R. Archer*[¶], Jessica. R. Hunt*[#], Joanne L. Yarker, Alessandro Riccombeni*[†], Annette S. Little, Nicola J. McCarthy and Jonathan D. Moore.

Horizon Discovery, 8100 Cambridge Research Park, Waterbeach, Cambridge, CB25 9TL, United Kingdom.

*Author correspondence to Benedict C.S. Cross (Benedict.Cross@horizondiscovery.com)

[¶]Current address: AstraZeneca, The Darwin Building, Milton Rd, Milton, Cambridge CB4 0FZ, United Kingdom

[#]Current address: CRUK MedImmune Alliance Lab, Portway Building, Cambridge CB21 6GS, United Kingdom

†Current address: DNAnexus, 1975 W El Camino Real #101, Mountain View, CA 94040, United States

FIGURE S1 | *Analysis of eHAP cell ploidy following lentivirus transduction*

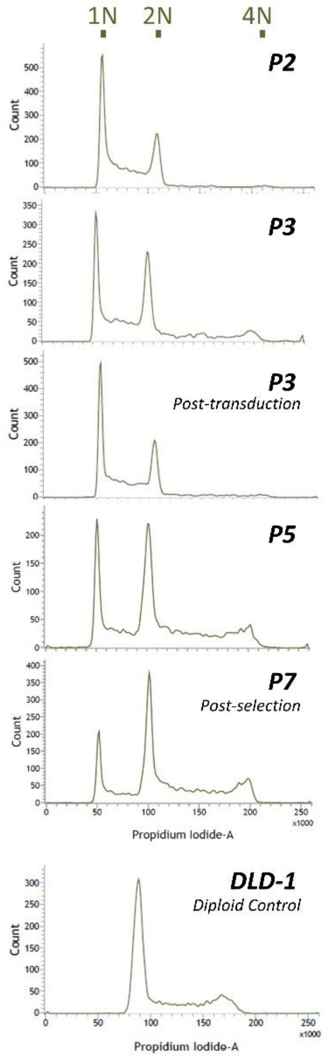

Supplementary Figure S1 | *Analysis of eHAP cell ploidy following lentivirus transduction.* Haploid eHAP cells were raised from cryopreservation and subjected to lentivirus transduction using a dummy sgRNA library. Transduced cells were then selected using puromycin and at each passage the cells were sampled and monitored for ploidy using propidium iodide-A staining and flow cytometry. The eHAP cells are largely haploid at early passage (P2, P3) and remain so throughout the early duration of the transduction process. Cells transition to diploid populations over the course of multiple passages. Importantly cells are largely haploid at the point of transduction and whilst the CRISPR-Cas9 editing event occurs.

FIGURE S2 | *CRISPR-Cas9 Screening in eHAP Haploid cells using two tracrRNA variants*

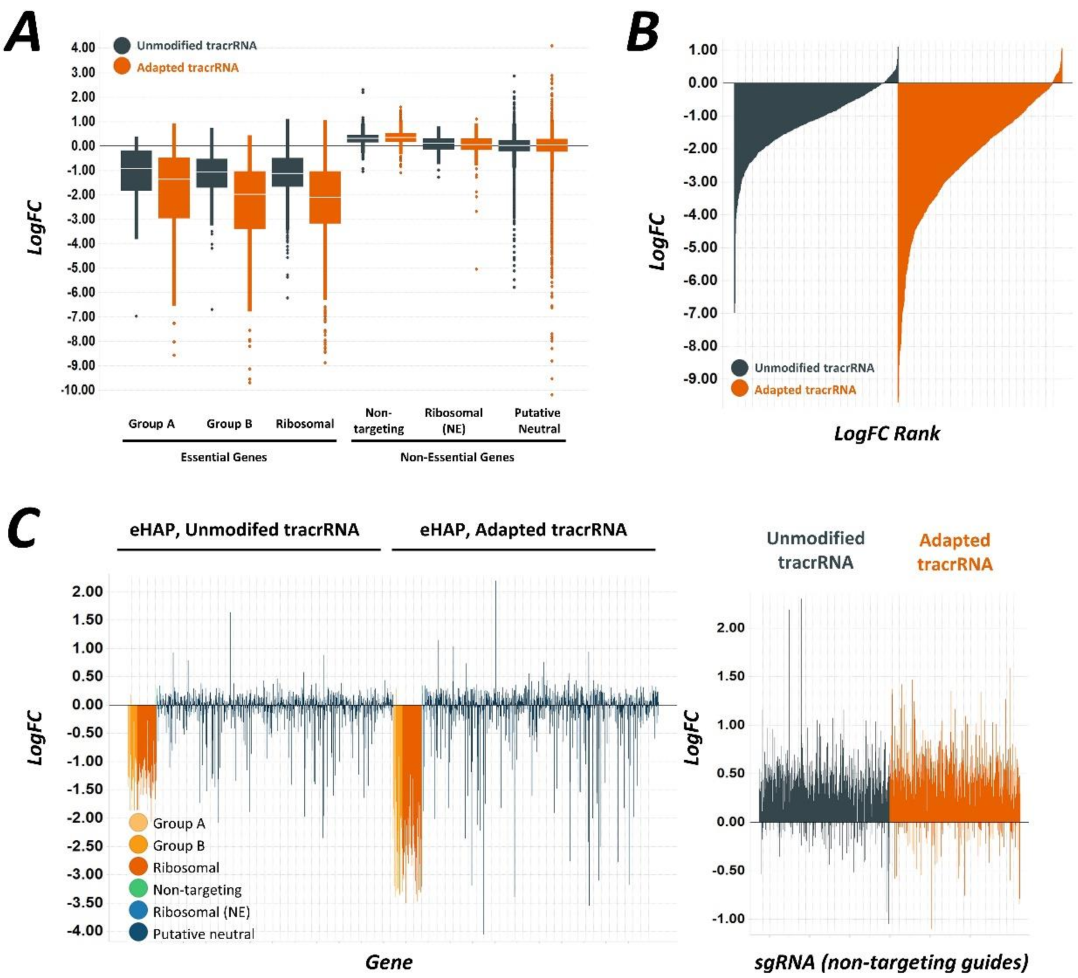

Supplementary Figure S2 | *CRISPR-Cas9 Screening in eHAP Haploid cells using two tracrRNA variants.* **A**. Box plots showing median guide LogFC for each group of genes and in each tracrRNA variant. **B**. Waterfall plots for the essential guides shown for each tracrRNA variant in eHAP cells. **C**. Mean LogFC drop-out rates for each gene in the eHAP screen for each tracrRNA variant, separated into groups. **D**. LogFC values for the non-targeting guides in each screen in A375 cells.

Table S1 | *Gene list and classification for sgRNA libraries used in this study.*

Gene_ID	Group
CDAN1	Group B
CDC16	Group B
GTF2B	Group B
HSPA9	Group B
PCNA	Group B
POLR2L	Group B
PREB	Group B
PSMA7	Group B
RPP21	Group B
SF3A3	Group B
SPC24	Group B
THAP1	Group B
TPT1	Group B
WDR74	Group B
SF3B3	Group B
PCF11	Group B
C1orf109	Group B
CDC123	Group B
ANAPC4	Group B
POLR2A	Group B
NT	Non-targeting
ABHD14B	Putative neutral
ADAMTS5	Putative neutral
ADPRHL2	Putative neutral
AIMP1	Putative neutral
AIP	Putative neutral
AJUBA	Putative neutral
AKR1B1	Putative neutral
AKR1C3	Putative neutral
ALPK2	Putative neutral
ALPK3	Putative neutral
ALS2CR8	Putative neutral
ANKRD12	Putative neutral
ANO7	Putative neutral
AP5Z1	Putative neutral
APAF1	Putative neutral
APEH	Putative neutral
APLP2	Putative neutral
APOBEC3B	Putative neutral
ARHGAP35	Putative neutral
ARHGAP4	Putative neutral
ARID3B	Putative neutral
ARID5B	Putative neutral

ASPA	Putative neutral
ASXL1	Putative neutral
ATF6B	Putative neutral
ATF7IP2	Putative neutral
ATOH8	Putative neutral
ATP1A3	Putative neutral
ATXN1	Putative neutral
ATXN1L	Putative neutral
ATXN7L3	Putative neutral
BANF2	Putative neutral
BARHL2	Putative neutral
BARX1	Putative neutral
BLCAP	Putative neutral
BLM	Putative neutral
BMPR2	Putative neutral
BNIP3	Putative neutral
BOK	Putative neutral
BRD2	Putative neutral
BRMS1L	Putative neutral
BSN	Putative neutral
C11orf30	Putative neutral
C11orf35	Putative neutral
C11orf48	Putative neutral
C11orf74	Putative neutral
C11orf96	Putative neutral
C12orf4	Putative neutral
C12orf42	Putative neutral
C12orf49	Putative neutral
C12orf65	Putative neutral
C12orf75	Putative neutral
C12orf76	Putative neutral
C14orf79	Putative neutral
C15orf39	Putative neutral
C15orf56	Putative neutral
C15orf60	Putative neutral
C16orf58	Putative neutral
C16orf78	Putative neutral
C17orf59	Putative neutral
C18orf25	Putative neutral
C19orf10	Putative neutral
C19orf18	Putative neutral
C19orf43	Putative neutral
C19orf44	Putative neutral
C19orf48	Putative neutral
C1orf101	Putative neutral
C1orf185	Putative neutral

C1orf210	Putative neutral
C1orf226	Putative neutral
C1orf27	Putative neutral
C20orf197	Putative neutral
C22orf15	Putative neutral
C22orf26	Putative neutral
C22orf28	Putative neutral
C2orf15	Putative neutral
C2orf43	Putative neutral
C2orf76	Putative neutral
C3orf38	Putative neutral
C3orf70	Putative neutral
C3orf80	Putative neutral
C4orf19	Putative neutral
C4orf27	Putative neutral
C4orf29	Putative neutral
C5orf34	Putative neutral
C5orf38	Putative neutral
C5orf42	Putative neutral
C6orf10	Putative neutral
C6orf120	Putative neutral
C6orf136	Putative neutral
C6orf70	Putative neutral
C7orf50	Putative neutral
C7orf60	Putative neutral
C8orf31	Putative neutral
C8orf34	Putative neutral
C8orf44	Putative neutral
C9orf116	Putative neutral
C9orf117	Putative neutral
C9orf129	Putative neutral
C9orf24	Putative neutral
C9orf50	Putative neutral
C9orf89	Putative neutral
C9orf96	Putative neutral
CACUL1	Putative neutral
CALCOCO1	Putative neutral
CAMK2N2	Putative neutral
CAMTA1	Putative neutral
CAND1	Putative neutral
CASP8AP2	Putative neutral
CBFA2T2	Putative neutral
CBFA2T3	Putative neutral
CBL	Putative neutral
CBR3	Putative neutral
CBX7	Putative neutral

CCDC110	Putative neutral
CCDC125	Putative neutral
CCDC132	Putative neutral
CCDC154	Putative neutral
CCDC169	Putative neutral
CCDC37	Putative neutral
CCDC69	Putative neutral
CCDC74B	Putative neutral
CCDC77	Putative neutral
CCDC80	Putative neutral
CCDC97	Putative neutral
CCND1	Putative neutral
CCNG1	Putative neutral
CCT5	Putative neutral
CDC25B	Putative neutral
CDC34	Putative neutral
CDK3	Putative neutral
CDK8	Putative neutral
CDKN1A	Putative neutral
CDKN1C	Putative neutral
CDKN2B	Putative neutral
CENPI	Putative neutral
CEP78	Putative neutral
CHD1L	Putative neutral
CHD9	Putative neutral
CHMP1A	Putative neutral
CHP2	Putative neutral
CIZ1	Putative neutral
CLEC3B	Putative neutral
CLIP1	Putative neutral
CLSTN1	Putative neutral
CMAS	Putative neutral
CMPK1	Putative neutral
COTL1	Putative neutral
CPSF3L	Putative neutral
CREBBP	Putative neutral
CREBRF	Putative neutral
CRTC3	Putative neutral
CRYAA	Putative neutral
CRYBA1	Putative neutral
CSF1R	Putative neutral
CSRNP2	Putative neutral
CSRP2	Putative neutral
CTCFL	Putative neutral
CTNND2	Putative neutral
CUL4A	Putative neutral

CXADR	Putative neutral
CXorf21	Putative neutral
CXorf23	Putative neutral
CXorf36	Putative neutral
DAZAP2	Putative neutral
DBX2	Putative neutral
DDX18	Putative neutral
DDX24	Putative neutral
DDX3X	Putative neutral
DDX4	Putative neutral
DEAF1	Putative neutral
DEDD	Putative neutral
DEDD2	Putative neutral
DFFB	Putative neutral
DGCR6	Putative neutral
DGCR8	Putative neutral
DIP2C	Putative neutral
DLX4	Putative neutral
DMBX1	Putative neutral
DNAJB4	Putative neutral
DNMT3L	Putative neutral
DOCK7	Putative neutral
DPEP3	Putative neutral
DPF2	Putative neutral
DRGX	Putative neutral
DROSHA	Putative neutral
DSCC1	Putative neutral
DUSP16	Putative neutral
DUSP18	Putative neutral
DUSP2	Putative neutral
DUSP5	Putative neutral
DYNC1I2	Putative neutral
DYNLT1	Putative neutral
DYNLT3	Putative neutral
DZIP1	Putative neutral
EDC4	Putative neutral
EGLN2	Putative neutral
EHF	Putative neutral
EID3	Putative neutral
ELK1	Putative neutral
EMC8	Putative neutral
ENC1	Putative neutral
EPHA10	Putative neutral
EPS15L1	Putative neutral
ERCC6L	Putative neutral
ERF	Putative neutral

ETHE1	Putative neutral
EYA3	Putative neutral
FADS1	Putative neutral
FAM102A	Putative neutral
FAM107B	Putative neutral
FAM109A	Putative neutral
FAM110A	Putative neutral
FAM114A1	Putative neutral
FAM149B1	Putative neutral
FAM155A	Putative neutral
FAM155B	Putative neutral
FAM167A	Putative neutral
FAM180A	Putative neutral
FAM181A	Putative neutral
FAM188B	Putative neutral
FAM196A	Putative neutral
FAM199X	Putative neutral
FAM19A4	Putative neutral
FAM208A	Putative neutral
FAM210B	Putative neutral
FAM222A	Putative neutral
FAM24A	Putative neutral
FAM35A	Putative neutral
FAM43B	Putative neutral
FAM46C	Putative neutral
FAM53A	Putative neutral
FAM5B	Putative neutral
FAM71E1	Putative neutral
FAM71F2	Putative neutral
FAM73A	Putative neutral
FAM76B	Putative neutral
FAM81A	Putative neutral
FAM83H	Putative neutral
FAM86A	Putative neutral
FAM90A1	Putative neutral
FAM91A1	Putative neutral
FANCC	Putative neutral
FBXO32	Putative neutral
FBXO40	Putative neutral
FBXO43	Putative neutral
FGF18	Putative neutral
FGFR4	Putative neutral
FOLH1	Putative neutral
FOSL2	Putative neutral
FOXD1	Putative neutral
FOXE1	Putative neutral

FOXI1	Putative neutral
FOXK2	Putative neutral
FOXO1	Putative neutral
FOXO3	Putative neutral
G3BP1	Putative neutral
GAS2L2	Putative neutral
GAS7	Putative neutral
GATA4	Putative neutral
GATAD2A	Putative neutral
GCAT	Putative neutral
GEM	Putative neutral
GHRHR	Putative neutral
GML	Putative neutral
GMNN	Putative neutral
GOLIM4	Putative neutral
GON4L	Putative neutral
GRB2	Putative neutral
GRHL2	Putative neutral
GRHL3	Putative neutral
GTF2F1	Putative neutral
GTF2I	Putative neutral
GTF2IRD1	Putative neutral
HDAC4	Putative neutral
HDGFRP3	Putative neutral
HELB	Putative neutral
HELQ	Putative neutral
HEPACAM2	Putative neutral
HES1	Putative neutral
HIST1H1B	Putative neutral
HIST1H2BM	Putative neutral
HIST3H3	Putative neutral
HLTF	Putative neutral
HMGCS1	Putative neutral
HMGN3	Putative neutral
HMGXB3	Putative neutral
HMX3	Putative neutral
HNF1A	Putative neutral
HNF1B	Putative neutral
HNRNPD	Putative neutral
HNRNPF	Putative neutral
HORMAD1	Putative neutral
HOXA1	Putative neutral
HOXA5	Putative neutral
HOXB13	Putative neutral
HOXB2	Putative neutral
HOXC12	Putative neutral

HOXC9	Putative neutral
HOXD1	Putative neutral
HOXD4	Putative neutral
HOXD9	Putative neutral
HSH2D	Putative neutral
HSPB3	Putative neutral
IDH3G	Putative neutral
IFI16	Putative neutral
IGFN1	Putative neutral
IGHMBP2	Putative neutral
IKZF2	Putative neutral
IL15RA	Putative neutral
IL1RN	Putative neutral
INVS	Putative neutral
IQGAP1	Putative neutral
IQSEC3	Putative neutral
IRAK1BP1	Putative neutral
IRAK2	Putative neutral
IRAK4	Putative neutral
IRF8	Putative neutral
IRX1	Putative neutral
ISL1	Putative neutral
ITGB3	Putative neutral
JUND	Putative neutral
KAT6A	Putative neutral
KAZN	Putative neutral
KCNH1	Putative neutral
KCTD13	Putative neutral
KDM4B	Putative neutral
KHDC3L	Putative neutral
KHDRBS2	Putative neutral
KIAA0101	Putative neutral
KIAA0146	Putative neutral
KIAA0195	Putative neutral
KIAA0556	Putative neutral
KIAA0753	Putative neutral
KIAA1211	Putative neutral
KIAA1407	Putative neutral
KIAA1430	Putative neutral
KIAA1549L	Putative neutral
KIAA1644	Putative neutral
KIAA1715	Putative neutral
KIAA2018	Putative neutral
KIF18A	Putative neutral
KIF18B	Putative neutral
KIF1A	Putative neutral

KIF25	Putative neutral
KLF6	Putative neutral
KLK10	Putative neutral
KLK6	Putative neutral
KLLN	Putative neutral
KPNA2	Putative neutral
KRT18	Putative neutral
LANCL2	Putative neutral
LARP6	Putative neutral
LARP7	Putative neutral
LEMD2	Putative neutral
LFNG	Putative neutral
LIN54	Putative neutral
LOC152586	Putative neutral
LOXL3	Putative neutral
LPIN3	Putative neutral
LRIF1	Putative neutral
LSMD1	Putative neutral
LYL1	Putative neutral
MAB21L2	Putative neutral
MACROD1	Putative neutral
MAEL	Putative neutral
MAFF	Putative neutral
MALT1	Putative neutral
MAP3K11	Putative neutral
MAP3K7	Putative neutral
MAPK8IP1	Putative neutral
MAST2	Putative neutral
MAST3	Putative neutral
MDM1	Putative neutral
MED13L	Putative neutral
MED15	Putative neutral
MED24	Putative neutral
MEIOB	Putative neutral
MID1IP1	Putative neutral
MKI67	Putative neutral
MLF1IP	Putative neutral
MLIP	Putative neutral
MLLT1	Putative neutral
MLLT3	Putative neutral
MMP2	Putative neutral
MNDA	Putative neutral
MPO	Putative neutral
MRO	Putative neutral
MTA2	Putative neutral
MTMR6	Putative neutral

MTUS1	Putative neutral
MXI1	Putative neutral
MYBL1	Putative neutral
MYH8	Putative neutral
MYO16	Putative neutral
MYOCD	Putative neutral
MYRF	Putative neutral
MYSM1	Putative neutral
NACA	Putative neutral
NACA2	Putative neutral
NACC2	Putative neutral
NAP1L2	Putative neutral
NAP1L5	Putative neutral
NDEL1	Putative neutral
NDRG2	Putative neutral
NEDD9	Putative neutral
NEK2	Putative neutral
NFATC1	Putative neutral
NFE2	Putative neutral
NFE2L3	Putative neutral
NFIB	Putative neutral
NGFRAP1	Putative neutral
NGRN	Putative neutral
NME2	Putative neutral
NOD2	Putative neutral
NOL4	Putative neutral
NOSTRIN	Putative neutral
NOX4	Putative neutral
NOX5	Putative neutral
NPAS2	Putative neutral
NPLOC4	Putative neutral
NPR1	Putative neutral
NQO2	Putative neutral
NR0B1	Putative neutral
NREP	Putative neutral
NRL	Putative neutral
NUDT12	Putative neutral
NUMB	Putative neutral
NUP210	Putative neutral
NXF3	Putative neutral
OAZ3	Putative neutral
ONECUT1	Putative neutral
OSBPL3	Putative neutral
OSR2	Putative neutral
OTX1	Putative neutral
OXR1	Putative neutral

PARD6A	Putative neutral
PARP12	Putative neutral
PAX9	Putative neutral
PDLIM2	Putative neutral
PDZD2	Putative neutral
PHF20	Putative neutral
PID1	Putative neutral
PIK3C2B	Putative neutral
PLD6	Putative neutral
PLEKHF1	Putative neutral
PLXNA1	Putative neutral
PMS2	Putative neutral
PNMA2	Putative neutral
POLB	Putative neutral
POLDIP2	Putative neutral
PON2	Putative neutral
POU3F1	Putative neutral
PPARG	Putative neutral
PPARGC1B	Putative neutral
PPIP5K1	Putative neutral
PPM1A	Putative neutral
PPP1R26	Putative neutral
PPP2R5C	Putative neutral
PPP2R5D	Putative neutral
PPP3CA	Putative neutral
PPP6C	Putative neutral
PPRC1	Putative neutral
PRDM8	Putative neutral
PRDX1	Putative neutral
PRKCA	Putative neutral
PRKD3	Putative neutral
PRKG2	Putative neutral
PRMT6	Putative neutral
PROP1	Putative neutral
PSMB8	Putative neutral
PSMC3IP	Putative neutral
PSME3	Putative neutral
PSME4	Putative neutral
PTMS	Putative neutral
PTOV1	Putative neutral
PYGO2	Putative neutral
RAB11FIP3	Putative neutral
RALGAPA1	Putative neutral
RALY	Putative neutral
RANBP17	Putative neutral
RAVER2	Putative neutral

RB1	Putative neutral
RBM34	Putative neutral
RBM38	Putative neutral
RBM42	Putative neutral
RCAN1	Putative neutral
RDH10	Putative neutral
RGS12	Putative neutral
RGS2	Putative neutral
RGS9	Putative neutral
RINT1	Putative neutral
RMDN3	Putative neutral
RNF10	Putative neutral
RNF14	Putative neutral
RNF168	Putative neutral
RNF6	Putative neutral
RPS4Y1	Putative neutral
RPS6KB2	Putative neutral
RSC1A1	Putative neutral
S100A12	Putative neutral
S100A6	Putative neutral
S100B	Putative neutral
SALL4	Putative neutral
SCGB1A1	Putative neutral
SCML4	Putative neutral
SENP6	Putative neutral
Sep-12	Putative neutral
Sep-14	Putative neutral
SERGEF	Putative neutral
SETD2	Putative neutral
SETD6	Putative neutral
SETD8	Putative neutral
SGK494	Putative neutral
SHOC2	Putative neutral
SHPRH	Putative neutral
SIM1	Putative neutral
SIT1	Putative neutral
SIX2	Putative neutral
SIX6	Putative neutral
SLC30A9	Putative neutral
SLFN11	Putative neutral
SMAD9	Putative neutral
SMC3	Putative neutral
SMYD3	Putative neutral
SNAP29	Putative neutral
SNX20	Putative neutral
SOHLH1	Putative neutral

SOHLH2	Putative neutral
SORBS1	Putative neutral
SORBS2	Putative neutral
SPAG8	Putative neutral
SPATA24	Putative neutral
SPEG	Putative neutral
SRC	Putative neutral
SRCAP	Putative neutral
SRRM4	Putative neutral
SRSF8	Putative neutral
SRY	Putative neutral
SSH3	Putative neutral
SSNA1	Putative neutral
ST6GALNAC4	Putative neutral
STAMBP	Putative neutral
STK17A	Putative neutral
STK24	Putative neutral
STK32C	Putative neutral
STMN1	Putative neutral
SUDS3	Putative neutral
SUGP1	Putative neutral
SYCE1L	Putative neutral
SYK	Putative neutral
SYN1	Putative neutral
SYNE3	Putative neutral
SYTL4	Putative neutral
TAF1C	Putative neutral
TAF5L	Putative neutral
TAOK3	Putative neutral
TBL1X	Putative neutral
TBP	Putative neutral
TBPL1	Putative neutral
TBRG4	Putative neutral
TBX1	Putative neutral
TBX10	Putative neutral
TBX20	Putative neutral
TCEANC2	Putative neutral
TCF7L1	Putative neutral
TCL1A	Putative neutral
TDRD3	Putative neutral
TEAD3	Putative neutral
TENM4	Putative neutral
TEX19	Putative neutral
TFDP2	Putative neutral
TGIF1	Putative neutral
TGS1	Putative neutral

TIGD4	Putative neutral
TIGD5	Putative neutral
TKTL1	Putative neutral
TMBIM6	Putative neutral
TMEM106B	Putative neutral
TMEM117	Putative neutral
TMEM125	Putative neutral
TMEM131	Putative neutral
TMEM136	Putative neutral
TMEM140	Putative neutral
TMEM143	Putative neutral
TMEM14A	Putative neutral
TMEM150B	Putative neutral
TMEM161A	Putative neutral
TMEM179B	Putative neutral
TMEM184A	Putative neutral
TMEM2	Putative neutral
TMEM203	Putative neutral
TMEM219	Putative neutral
TMEM232	Putative neutral
TMEM245	Putative neutral
TMEM247	Putative neutral
TMEM42	Putative neutral
TMEM5	Putative neutral
TMEM50A	Putative neutral
TMEM53	Putative neutral
TMEM60	Putative neutral
TMEM63A	Putative neutral
TMEM65	Putative neutral
TMEM81	Putative neutral
TMEM87A	Putative neutral
TMEM8B	Putative neutral
TMEM97	Putative neutral
TNFRSF1B	Putative neutral
TNKS	Putative neutral
TNNI2	Putative neutral
TOP1MT	Putative neutral
TOP3B	Putative neutral
TOR1A	Putative neutral
TOR1AIP1	Putative neutral
TP53BP1	Putative neutral
TPRKB	Putative neutral
TPRX1	Putative neutral
TREX2	Putative neutral
TRIM47	Putative neutral
TRIM66	Putative neutral

TRIO	Putative neutral
TSC22D3	Putative neutral
TSHZ3	Putative neutral
TSPYL1	Putative neutral
TSPYL2	Putative neutral
TSSK6	Putative neutral
TTBK1	Putative neutral
TTBK2	Putative neutral
TTLL10	Putative neutral
TTYH1	Putative neutral
TUB	Putative neutral
TUBB4A	Putative neutral
TULP3	Putative neutral
TXK	Putative neutral
TXN2	Putative neutral
TYK2	Putative neutral
U2AF1L4	Putative neutral
UAP1	Putative neutral
UBD	Putative neutral
UBE2D2	Putative neutral
UBE2E3	Putative neutral
UBP1	Putative neutral
UFL1	Putative neutral
UHRF2	Putative neutral
ULK3	Putative neutral
UNKL	Putative neutral
URGCP	Putative neutral
USB1	Putative neutral
USH1C	Putative neutral
USO1	Putative neutral
USP44	Putative neutral
UTP6	Putative neutral
VAX2	Putative neutral
VGLL3	Putative neutral
VIPR1	Putative neutral
WDR26	Putative neutral
WDR27	Putative neutral
WDR31	Putative neutral
WDR45B	Putative neutral
WDR52	Putative neutral
WDR54	Putative neutral
WDR60	Putative neutral
WIZ	Putative neutral
WWC1	Putative neutral
XRCC5	Putative neutral
YEATS2	Putative neutral

YPEL1	Putative neutral
YWHAB	Putative neutral
ZBTB24	Putative neutral
ZBTB3	Putative neutral
ZBTB34	Putative neutral
ZBTB4	Putative neutral
ZC2HC1C	Putative neutral
ZCCHC3	Putative neutral
ZCCHC6	Putative neutral
ZCCHC9	Putative neutral
ZDHHC1	Putative neutral
ZDHHC13	Putative neutral
ZDHHC14	Putative neutral
ZDHHC16	Putative neutral
ZDHHC20	Putative neutral
ZDHHC3	Putative neutral
ZDHHC6	Putative neutral
ZFAT	Putative neutral
ZFP14	Putative neutral
ZFP92	Putative neutral
ZFR	Putative neutral
ZFY	Putative neutral
ZGPAT	Putative neutral
ZHX1	Putative neutral
ZIC2	Putative neutral
ZIC4	Putative neutral
ZKSCAN1	Putative neutral
ZMYM6	Putative neutral
ZMYND19	Putative neutral
ZNF107	Putative neutral
ZNF140	Putative neutral
ZNF165	Putative neutral
ZNF185	Putative neutral
ZNF248	Putative neutral
ZNF264	Putative neutral
ZNF266	Putative neutral
ZNF281	Putative neutral
ZNF34	Putative neutral
ZNF354A	Putative neutral
ZNF382	Putative neutral
ZNF394	Putative neutral
ZNF395	Putative neutral
ZNF432	Putative neutral
ZNF444	Putative neutral
ZNF468	Putative neutral
ZNF470	Putative neutral

ZNF48	Putative neutral
ZNF506	Putative neutral
ZNF507	Putative neutral
ZNF516	Putative neutral
ZNF530	Putative neutral
ZNF532	Putative neutral
ZNF562	Putative neutral
ZNF570	Putative neutral
ZNF573	Putative neutral
ZNF583	Putative neutral
ZNF585A	Putative neutral
ZNF613	Putative neutral
ZNF621	Putative neutral
ZNF628	Putative neutral
ZNF652	Putative neutral
ZNF668	Putative neutral
ZNF670	Putative neutral
ZNF703	Putative neutral
ZNF710	Putative neutral
ZNF749	Putative neutral
ZNF76	Putative neutral
ZNF771	Putative neutral
ZNF780A	Putative neutral
ZNF804A	Putative neutral
ZNF81	Putative neutral
ZNF813	Putative neutral
ZNF821	Putative neutral
ZNF827	Putative neutral
ZNF860	Putative neutral
ZNF93	Putative neutral
ZSCAN5A	Putative neutral
ZUFSP	Putative neutral
ZXDC	Putative neutral
RPL39	Ribosomal
RPL41	Ribosomal
RPL38	Ribosomal
RPS26	Ribosomal
RPL17	Ribosomal
RPL10A	Ribosomal
RPL12	Ribosomal
RPL13	Ribosomal
RPL13A	Ribosomal
RPL14	Ribosomal
RPL21	Ribosomal
RPL22	Ribosomal
RPL23	Ribosomal

RPL23A	Ribosomal
RPL24	Ribosomal
RPL27	Ribosomal
RPL27A	Ribosomal
RPL28	Ribosomal
RPL29	Ribosomal
RPL30	Ribosomal
RPL31	Ribosomal
RPL32	Ribosomal
RPL34	Ribosomal
RPL35	Ribosomal
RPL35A	Ribosomal
RPL36	Ribosomal
RPL37A	Ribosomal
RPL5	Ribosomal
RPL7	Ribosomal
RPL7A	Ribosomal
RPL7L1	Ribosomal
RPL8	Ribosomal
RPS14	Ribosomal
RPS15	Ribosomal
RPS15A	Ribosomal
RPS18	Ribosomal
RPS19	Ribosomal
RPS2	Ribosomal
RPS20	Ribosomal
RPS23	Ribosomal
RPS24	Ribosomal
RPS25	Ribosomal
RPS27	Ribosomal
RPS29	Ribosomal
RPS3	Ribosomal
RPS4X	Ribosomal
RPS5	Ribosomal
RPS6	Ribosomal
RPS7	Ribosomal
RPS8	Ribosomal
RPS9	Ribosomal
RPL26	Ribosomal
RPL37	Ribosomal
RPL15	Ribosomal
RPS28	Ribosomal
RPS21	Ribosomal
RPL6	Ribosomal
RPL9	Ribosomal
RPL10	Ribosomal

RPL11	Ribosomal
RPL3	Ribosomal
RPL4	Ribosomal
RPL39L	Ribosomal_NE
RPL10L	Ribosomal_NE
RPL22L1	Ribosomal_NE
RPS27L	Ribosomal_NE
RPS4Y1	Ribosomal_NE
RPS4Y2	Ribosomal_NE
RPL3L	Ribosomal_NE
RPL26L1	Ribosomal_NE
CCDC84	Group A
HCFC1	Group A
MED30	Group A
MYC	Group A
POLR2I	Group A
SNRNP25	Group A
TINF2	Group A
TRIB1	Group A
CDCA8	Group A
SON	Group A
